# Association of comorbidity burden with initial treatment allocation in older women with gynecologic cancer: a retrospective cohort study

**DOI:** 10.3389/fmed.2026.1802265

**Published:** 2026-07-28

**Authors:** Lin Tang, Yuhang Liu, Bin Chen, Hong Zou, Yongqiang Yang, Xingyu Sun, Lijun Zhong

**Affiliations:** 1 Department of Gynecology, The Affiliated Traditional Chinese Medicine Hospital, Southwest Medical University, Luzhou, Sichuan, China; 2 Southwest Medical University, Luzhou, Sichuan, China

**Keywords:** comorbidity, gynecologic cancer, older patients, predictive modeling, treatment allocation

## Abstract

**Background:**

Comorbidity is a key determinant of treatment decisions in older cancer patients; however, its role in shaping initial treatment allocation among older women with gynecologic malignancies remains insufficiently characterized. We aimed to evaluate the association between comorbidity burden and treatment selection and to develop a clinically interpretable predictive model.

**Methods:**

We retrospectively analyzed 972 women aged ≥65 years with newly diagnosed cervical, ovarian, or endometrial cancer treated at a tertiary hospital in Southwest China between 2019 and 2024. Disease-specific guideline-concordant standard treatment was defined as curative-intent initial treatment appropriate for tumor type and FIGO stage, including surgery, platinum-based chemotherapy, concurrent chemoradiotherapy, brachytherapy, or combined-modality treatment when indicated. Multivariable logistic regression was used to identify independent predictors of disease-specific guideline-concordant standard treatment, and a predictive model was developed and internally validated with assessments of discrimination, calibration, and clinical utility.

**Results:**

Of the 972 patients, 63.7% received disease-specific guideline-concordant standard treatment. Higher comorbidity burden [Charlson Comorbidity Index (CCI) ≥ 4], ECOG performance status ≥2, age ≥75 years, and pulmonary disease were independently associated with lower odds of receiving disease-specific guideline-concordant standard treatment (all *p* < 0.05). In contrast, higher body mass index and serum albumin levels were associated with higher odds of receiving disease-specific guideline-concordant standard treatment. The final model demonstrated strong internally validated discriminatory performance (AUC = 0.933, 95% CI: 0.917–0.948), good calibration, and meaningful clinical utility across a wide range of decision thresholds.

**Conclusion:**

In this pooled real-world observational cohort of older women with cervical, ovarian, or endometrial cancer, comorbidity burden was independently associated with receipt of disease-specific guideline-concordant standard treatment. The internally validated host-factor-oriented model may support individualized treatment discussions as an adjunct to multidisciplinary assessment, but it should not be interpreted as a tumor-specific treatment algorithm or as a replacement for disease-specific guideline-based decision-making. Further external validation and tumor-specific prospective studies are needed before broader clinical implementation.

## Introduction

The global rise in life expectancy has contributed to a growing population of older women diagnosed with gynecologic malignancies, including cervical, ovarian, and endometrial cancer (1). Among this demographic, treatment decisions are often complicated by the presence of multiple chronic conditions and age-related physiological decline (2, 3). While age and performance status are widely acknowledged as key factors in oncologic decision-making, the specific influence of comorbidity burden on initial treatment selection in gynecologic cancers remains insufficiently characterized—particularly within real-world clinical practice and across Asian patient populations. Recent studies from East Asia suggest that older women with ovarian or endometrial cancer frequently present with high levels of multimorbidity, which is associated with a lower likelihood of receiving multimodal or guideline-concordant cancer treatment, even after consideration of chronological age or performance status (4–6). Comorbidity—defined as the coexistence of one or more chronic diseases—differs conceptually from frailty or functional status. While frailty reflects a multidimensional syndrome of diminished physiological reserve and vulnerability, and functional status refers to current performance capacity, comorbidity remains a distinct construct with independent prognostic relevance in treatment outcomes among older adults (7, 8). Instruments such as the Charlson Comorbidity Index (CCI) and its age-adjusted variant (ACCI) have been extensively validated in oncology and consistently demonstrate strong prognostic value for mortality and treatment-related outcomes, independent of tumor characteristics or therapeutic modality (9, 10). Numerous studies have shown that higher comorbidity scores—most commonly assessed using the CCI—are associated with a lower probability of receiving cancer-directed or guideline-concordant treatment, and with poorer short- and long-term survival outcomes across malignancy types (11, 12). However, these findings have been derived predominantly from Western cohorts and may have limited applicability to healthcare infrastructures, treatment access patterns, and sociocultural dynamics in China and other low- and middle-income countries (13).

Importantly, current clinical guidelines do not provide explicit thresholds or decision-making frameworks that incorporate comorbidity burden into treatment planning for older women with gynecologic cancers (14). In the absence of standardized, evidence-based tools, clinicians often rely on subjective judgment, which can result in under-treatment, overtreatment, or significant variability in care across institutions and providers (15). Predictive models that integrate objective clinical and laboratory indicators—including comorbidity burden (e.g., via ACE-27), performance status (e.g., ECOG), nutritional markers (e.g., serum albumin or the HALP index), and tumor-related features—may offer valuable decision support. Nevertheless, such models remain rare for this patient group, and existing tools often focus on surgical risk alone or emphasize isolated domains rather than adopting a comprehensive host–tumor framework (16, 17).

Furthermore, many studies either pool data across different gynecologic cancer types or systematically exclude older patients, thereby failing to capture meaningful differences in treatment feasibility, toxicity, and disease trajectory between cervical, endometrial, and ovarian cancers (18). This practice limits our understanding of how comorbidity burden differentially influences treatment selection across tumor types. Notably, few predictive tools have been developed or validated specifically for Chinese oncology populations—a critical gap given China’s accelerating demographic shift toward an aging society and the increasing strain it places on oncology care systems (19).

To address these gaps, we conducted a retrospective cohort study of nearly 1,000 older women with newly diagnosed cervical, ovarian, or endometrial cancer treated at a tertiary referral center in Southwest China. Because comorbidity burden, functional status, and nutritional reserve are common host-related factors considered across geriatric oncology practice, the pooled cohort was designed to evaluate shared patient-level determinants of initial treatment allocation rather than to imply that these malignancies have identical treatment paradigms. Our objectives were twofold: (1) to systematically assess the independent association between comorbidity burden and the likelihood of receiving disease-specific, guideline-concordant standard treatment; and (2) to develop and internally validate a host-factor-oriented predictive model to support individualized treatment planning as a complement to, rather than a substitute for, tumor-specific clinical decision-making.

## Methods

### Study design and population

This retrospective cohort study was conducted at a tertiary academic medical center in Southwest China. Patients aged 65 years or older with a newly diagnosed, histologically confirmed primary gynecologic malignancy—including cervical, ovarian, or endometrial cancer—between March 1, 2019, and June 30, 2024, were eligible for inclusion. These three malignancies were analyzed together because they represent the major gynecologic cancers encountered in routine practice at our institution and share a common clinical challenge in older patients: evaluating whether the patient’s comorbidity burden, functional status, and physiological reserve are compatible with disease-specific standard treatment. The pooled analysis was therefore intended to assess common host-related determinants of treatment allocation, while disease-related heterogeneity was addressed by incorporating tumor type and FIGO stage into the analytic framework and by conducting more granular diagnosis-based sensitivity analyses. Cases were identified through the institutional cancer registry and electronic medical records (EMRs) using ICD-10 codes (C53, C56, and C54). Inclusion required receipt of both diagnosis and initial treatment at the study center and the availability of complete baseline clinical data, including demographic information, laboratory findings, tumor characteristics, comorbid conditions, and documented treatment modality. Patients were excluded if they had recurrent gynecologic cancer, any other malignancy within the preceding 5 years, metastatic disease of non-gynecologic origin (e.g., Krukenberg tumors), incomplete key variables necessary for modeling, or were managed primarily outside the institution or under protocol-based investigational therapy. These exclusions were applied to maintain a cohort of older women with newly diagnosed primary gynecologic malignancies in whom the outcome reflected initial treatment allocation. Patients with recurrent disease or metastatic malignancies were excluded because their treatment decisions are often shaped by prior surgery, chemotherapy, radiotherapy, treatment response, treatment-related toxicity, disease-free interval, and accumulated organ dysfunction, and because they may represent a more heterogeneous population with advanced illness and higher medical complexity. To reduce selection bias, eligibility screening and data abstraction were independently conducted by two trained investigators according to a prespecified protocol, with discrepancies resolved through adjudication by a senior gynecologic oncologist. The study protocol was approved by the Institutional Review Board of the participating hospital. Given the retrospective nature and use of anonymized data, the requirement for written informed consent was waived.

### Tumor assessment and diagnostic consistency

Tumor staging was determined according to the 2009 or 2018 FIGO criteria (as appropriate by cancer type and diagnosis year), based on operative findings, imaging, and histopathologic assessment. All pathological diagnoses and staging evaluations were performed or reviewed by board-certified gynecologic oncologists and institutional pathologists to ensure consistency. Patients with unclear staging or discordant diagnostic data were excluded from analysis.

### Treatment decision-making and pre-treatment evaluation

Initial treatment strategies were determined by the attending gynecologic oncologist. In complex or high-risk cases, decisions were made through multidisciplinary tumor board (MDT) discussions involving surgical oncologists, medical oncologists, geriatricians, and anesthesiologists. Disease-specific guideline-concordant standard treatment was defined as curative-intent initial treatment appropriate for tumor type and FIGO stage, including surgery, platinum-based chemotherapy, concurrent chemoradiotherapy, brachytherapy, or combined-modality treatment when indicated, based on contemporary disease-specific clinical guidelines for cervical, ovarian, and endometrial cancer (20–22). Non-standard treatment was defined as deviation from disease-specific guideline-concordant standard treatment, including hormonal therapy alone, palliative treatment, best supportive care, or treatment de-escalation primarily because of medical complexity, poor functional status, or patient/family preference.

For cervical cancer, disease-specific guideline-concordant standard treatment was determined primarily according to FIGO stage and radiotherapy feasibility, rather than surgical considerations alone. For selected early-stage cervical cancer, surgery-based curative treatment was considered guideline-concordant when appropriate. For locally advanced cervical cancer, standard treatment primarily consisted of definitive concurrent chemoradiotherapy, including external-beam radiotherapy, platinum-based chemotherapy, and brachytherapy when feasible (23, 24). Therefore, cervical cancer patients were not classified as receiving non-standard treatment solely because they did not undergo surgery. For older patients with cervical cancer, treatment feasibility was assessed not only in terms of surgical fitness but also in terms of radiotherapy access, feasibility of brachytherapy, ability to tolerate concurrent chemotherapy, baseline organ function, ECOG performance status, comorbidity burden, and the anticipated ability to complete the full radiotherapy course.

For endometrial cancer, disease-specific guideline-concordant standard treatment was also determined according to FIGO stage, histologic subtype, tumor grade, and treatment feasibility. For most patients with early-stage endometrial cancer, standard treatment consisted of hysterectomy-based surgery, generally including total hysterectomy with bilateral salpingo-oophorectomy, with lymph node assessment or sentinel lymph node mapping when indicated. A minimally invasive surgical approach was considered when clinically feasible, particularly in patients with apparent uterine-confined disease. Adjuvant radiotherapy, chemotherapy, hormonal therapy, or combined-modality treatment was considered guideline-concordant when indicated by disease stage, histology, and risk profile.

For ovarian cancer, disease-specific guideline-concordant standard treatment included primary cytoreductive surgery followed by platinum-based chemotherapy, or neoadjuvant platinum-based chemotherapy followed by interval cytoreductive surgery when primary surgery was considered infeasible or unsafe. The choice between primary cytoreductive surgery and neoadjuvant chemotherapy was made through routine clinical assessment and, when appropriate, multidisciplinary discussion, taking into account ECOG performance status, comorbidity burden, nutritional reserve, anesthetic risk, imaging-based tumor extent, and the anticipated feasibility of optimal cytoreduction. However, standardized ovarian cancer-specific surgical risk scores, such as laparoscopic morbidity-prediction scores or formal resectability scoring systems, were not routinely documented in the electronic medical records during the study period and therefore were not included as model predictors.

Comprehensive geriatric assessment (CGA), G8 screening, or other formalized frailty tools were not routinely applied during the study period. Therefore, frailty could not be directly quantified in this retrospective dataset. Instead, functional status was assessed by clinicians using the ECOG performance status scale, which was consistently documented in all eligible patients and included as a pragmatic surrogate for treatment fitness. For ovarian cancer patients, ECOG performance status, comorbidity burden, nutritional reserve, and routine preoperative assessment were used clinically to inform surgical feasibility and the choice between primary cytoreductive surgery and neoadjuvant chemotherapy.

### Variables and data quality control

The primary outcome was the receipt of disease-specific guideline-concordant standard treatment. Predictor variables included age, BMI, serum albumin, tumor type, FIGO stage, ECOG performance status (PS), and comorbidity burden. Comorbidities were identified using clinical diagnosis codes and confirmed through manual medical record review. Comorbidity assessment included both conditions contributing to the Charlson Comorbidity Index (CCI) and additional individual comorbidities of clinical interest. The CCI was used to quantify overall comorbidity burden and was categorized as 0–1 (low), 2–3 (moderate), and ≥4 (high), consistent with previous cancer-related studies using Charlson comorbidity indices in older or oncologic populations (10, 25). Individual comorbidities, including hypertension, diabetes mellitus, coronary artery disease, chronic pulmonary disease including COPD, cerebrovascular disease, and chronic kidney disease, were extracted separately for descriptive analyses; clinically relevant conditions were also considered for inclusion in the multivariable model. All data fields were cross-validated with institutional registries. Missing data were less than 2% across all variables; specifically, fewer than 20 patients, representing approximately 1.8% of the cohort, had missing baseline predictors and were excluded from multivariable analyses using complete-case analysis.

To preserve patient confidentiality, all data were anonymized prior to analysis. No personal identifiers were collected, and results are presented only in aggregate form. The study complied with the Declaration of Helsinki and applicable data protection regulations.

### Sample size considerations

Although no formal *a priori* sample size calculation was performed because of the retrospective design, the sample size was considered adequate for multivariable modeling. Among 972 eligible patients, 619 received disease-specific guideline-concordant standard treatment and 353 received non-standard, de-escalated, or palliative treatment, providing a sufficient number of outcome events relative to the number of candidate predictors included in the final logistic regression model. Internal validation using bootstrap resampling with 1,000 repetitions was performed to reduce optimism in model performance estimates.

### Statistical analysis

**Figure 1:**
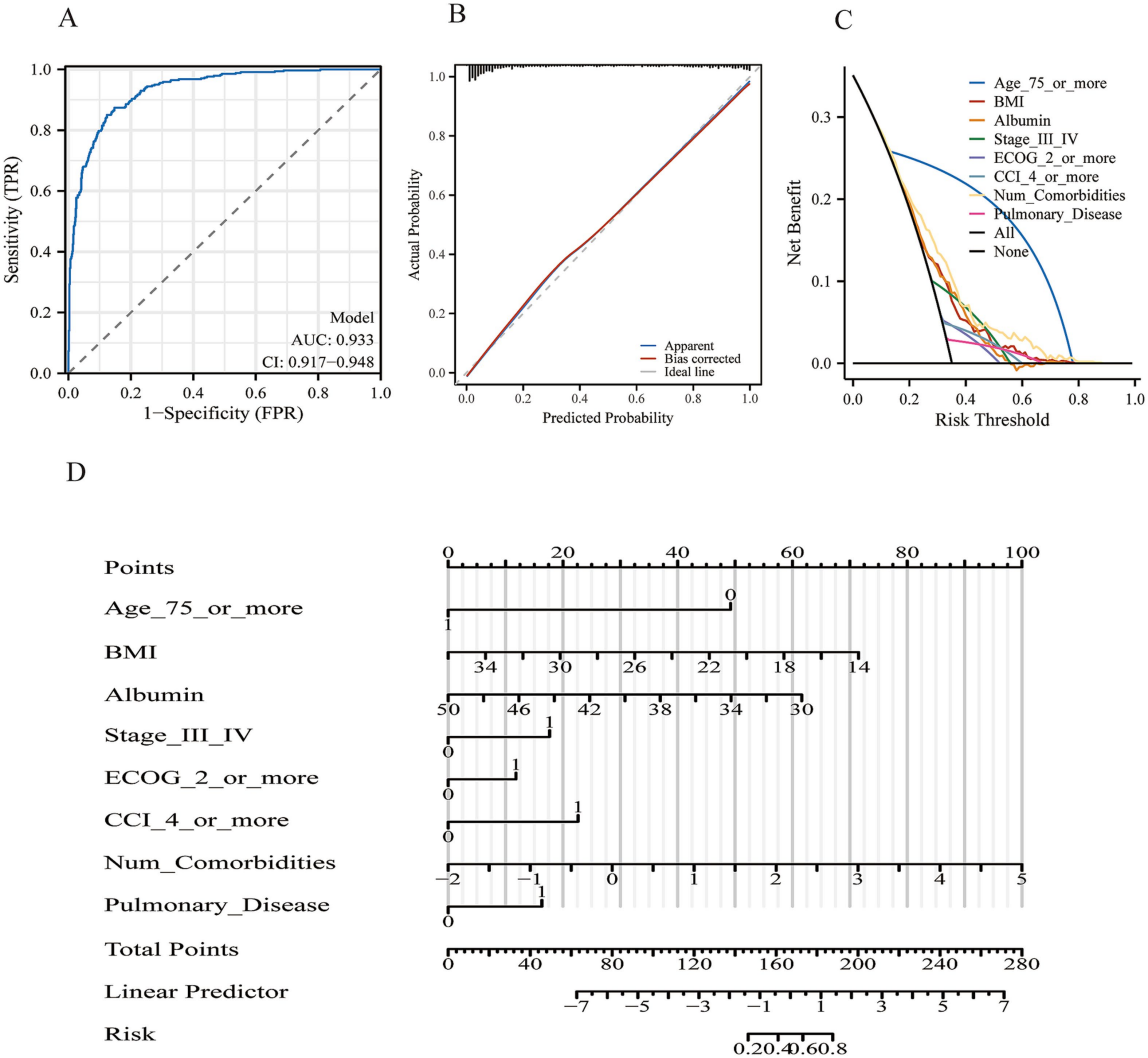
Performance evaluation of the predictive model for disease-specific guideline-concordant standard treatment in older gynecologic oncology patients. **(A)** Receiver Operating Characteristic (ROC) curve of the final model. The area under the curve (AUC) was 0.933 (95% CI: 0.917–0.948), indicating excellent discriminative ability. **(B)** Calibration plot of the predictive model. The apparent curve (blue) and bias-corrected curve (red) closely aligned with the ideal 45° reference line (gray), suggesting good model calibration. **(C)** Decision Curve Analysis (DCA) for net clinical benefit across a range of risk thresholds. The model showed superior net benefit compared to treating all or none, especially in the 10–70% threshold range. **(D)** Nomogram constructed from the final logistic regression model. Each predictor was assigned a point value, and total points were translated into the predicted probability of receiving disease-specific guideline-concordant standard treatment.

Descriptive statistics were used to summarize baseline characteristics of the study population (Table 1). Continuous variables were compared between the standard and non-standard treatment groups using the independent samples t test or the Mann–Whitney U test, as appropriate, while categorical variables were compared using the chi-square test (Table 2). To identify independent factors associated with receipt of disease-specific guideline-concordant standard treatment, a multivariable logistic regression model was constructed (Table 3). Candidate variables were selected based on clinical relevance and univariate associations (*p* < 0.10). Given the inherent heterogeneity among cervical, ovarian, and endometrial cancers, tumor type and FIGO stage were considered disease-related adjustment variables in the analytic framework to reduce confounding by disease-specific treatment pathways. The CCI category and the number of comorbidities were retained concurrently to capture complementary dimensions of comorbidity burden, reflecting weighted disease severity and overall disease count, respectively. Multicollinearity among predictors was assessed using variance inflation factors (VIFs), and all VIF values were <2, which was below commonly used conservative thresholds for problematic multicollinearity (26, 27). Model discrimination was evaluated using the area under the receiver operating characteristic curve (AUC), and calibration was assessed with the Hosmer–Lemeshow goodness-of-fit test and calibration plots. Internal validation was performed using bootstrap resampling with 1,000 repetitions to account for potential optimism in model performance estimates. Trends in treatment allocation across Charlson Comorbidity Index (CCI) categories were evaluated using the Cochran−Armitage trend test (Table 4). A nomogram was constructed based on the final model coefficients, and decision curve analysis (DCA) was conducted to assess clinical utility across a range of threshold probabilities (Figures 1C,D). Sensitivity analyses were performed to assess the robustness of the associations between comorbidity burden and receipt of disease-specific guideline-concordant standard treatment across tumor types and alternative comorbidity thresholds. These included (1) tumor type–stratified analyses for cervical, ovarian, and endometrial cancer, and (2) application of an alternative CCI cut-off (≥3 vs. ≥4). Within each tumor-specific stratum, higher comorbidity burden and impaired ECOG performance status were consistently associated with a lower likelihood of receiving disease-specific guideline-concordant standard treatment, with the magnitude of association varying by cancer type. Due to smaller sample sizes in each stratum, these analyses are presented as exploratory and summarized in Supplementary Table S1, with the text now explicitly referencing the table to improve clarity and accessibility of the results. All statistical analyses were conducted using R software (version 4.3.1). Two-sided *p* values < 0.05 were considered statistically significant.

**Table 1:** Baseline characteristics of the study population (*N* = 972).

Variable	Total (*N* = 972)
Demographics	
Age, mean ± SD, years	73.4 ± 5.1
Age group, *n* (%)	
65–69 years	342 (35.2)
70–74 years	301 (31.0)
≥75 years	329 (33.8)
BMI, mean ± SD, kg/m^2^	23.2 ± 3.8
Serum albumin, mean ± SD, g/L	37.1 ± 4.2
Tumor-related factors	
Cancer type, *n* (%)	
Cervical cancer	332 (34.1)
Ovarian cancer	289 (29.7)
Endometrial cancer	351 (36.1)
FIGO stage, *n* (%)	
I–II	473 (48.7)
III–IV	499 (51.3)
Comorbidity and functional status	
Charlson Comorbidity Index (CCI), *n* (%)	
0–1	235 (24.2)
2–3	428 (44.0)
≥4	309 (31.8)
Number of comorbidities, mean ± SD	2.9 ± 1.4
Common comorbidities, *n* (%)	
Hypertension	637 (65.5)
Diabetes mellitus	398 (40.9)
Coronary artery disease	213 (21.9)
Chronic pulmonary disease, including COPD	176 (18.1)
Cerebrovascular disease	154 (15.8)
Chronic kidney disease	108 (11.1)
ECOG performance status, *n* (%)	
0–1	591 (60.8)
≥2	381 (39.2)
Initial treatment allocation	
Disease-specific guideline-concordant standard treatment	619 (63.7)
Non-standard, de-escalated, or palliative treatment	353 (36.3)

SD, standard deviation; BMI, body mass index; FIGO, International Federation of Gynecology and Obstetrics; CCI, Charlson Comorbidity Index; ECOG, Eastern Cooperative Oncology Group; COPD, chronic obstructive pulmonary disease. Disease-specific guideline-concordant standard treatment was defined as curative-intent initial treatment appropriate for tumor type and FIGO stage, including surgery, platinum-based chemotherapy, concurrent chemoradiotherapy, brachytherapy, or combined-modality treatment when indicated. Non-standard, de-escalated, or palliative treatment included hormonal therapy alone, palliative treatment, best supportive care, or treatment de-escalation primarily because of medical complexity, poor functional status, or patient/family preference.

**Table 2:** Comparison of tumor, functional, and comorbidity characteristics between treatment allocation groups.

Variable	Disease-specific guideline-concordant standard treatment (*n* = 619)	Non-standard, de-escalated, or palliative treatment (*n* = 353)	*p*-value
Tumor characteristics			
Cancer type, *n* (%)			0.031^b^
Cervical cancer	222 (35.9)	110 (31.2)	
Ovarian cancer	197 (31.8)	92 (26.1)	
Endometrial cancer	200 (32.3)	151 (42.8)	
FIGO stage III–IV, *n* (%)	296 (47.8)	203 (57.5)	0.002^b^
Functional and comorbidity status			
ECOG PS ≥ 2, *n* (%)	160 (25.8)	221 (62.6)	<0.001^b^
CCI, median (IQR)	2 (1–3)	3 (2–4)	<0.001^c^
CCI ≥ 4, *n* (%)	142 (22.9)	167 (47.3)	<0.001^b^
Number of comorbidities, mean ± SD	2.5 ± 1.2	3.5 ± 1.6	<0.001^a^
Common comorbidities, *n* (%)			
Hypertension	373 (60.3)	264 (74.8)	<0.001^b^
Diabetes mellitus	222 (35.9)	176 (49.9)	<0.001^b^
Coronary artery disease	114 (18.4)	99 (28.0)	<0.001^b^
Pulmonary disease, including COPD	91 (14.7)	85 (24.1)	<0.001^b^
Cerebrovascular disease	81 (13.1)	73 (20.7)	0.002^b^

SD, standard deviation; IQR, interquartile range; FIGO, International Federation of Gynecology and Obstetrics; ECOG PS, Eastern Cooperative Oncology Group performance status; CCI, Charlson Comorbidity Index; COPD, chronic obstructive pulmonary disease. Disease-specific guideline-concordant standard treatment was defined as curative-intent initial treatment appropriate for tumor type and FIGO stage, including surgery, platinum-based chemotherapy, concurrent chemoradiotherapy, brachytherapy, or combined-modality treatment when indicated.

a Independent samples *t*-test.

b Chi-square test.

c Mann–Whitney U test.

**Table 3:** Multivariable logistic regression analysis of factors associated with receipt of disease-specific guideline-concordant standard treatment.

Variable	aOR	95% CI	*p*-value
Demographic and nutritional factors			
Age ≥75 years (ref: <75 years)	0.56	0.39–0.81	0.002**
BMI, per 1 kg/m^2^ increase	1.07	1.02–1.13	0.009*
Serum albumin, per 1 g/L increase	1.10	1.05–1.16	<0.001***
Tumor and functional status			
FIGO stage III–IV (ref: I–II)	0.73	0.55–0.97	0.031*
ECOG PS ≥ 2 (ref: 0–1)	0.39	0.28–0.54	<0.001***
Comorbidity burden			
CCI ≥ 4 (ref: <4)	0.52	0.38–0.72	<0.001***
Number of comorbidities, per 1 disease	0.82	0.75–0.91	<0.001***
Pulmonary disease (ref: no)	0.69	0.47–0.99	0.043*

aOR, adjusted odds ratio; CI, confidence interval; BMI, body mass index; FIGO, International Federation of Gynecology and Obstetrics; ECOG PS, Eastern Cooperative Oncology Group performance status; CCI, Charlson Comorbidity Index. The dependent variable was receipt of disease-specific guideline-concordant standard treatment. Reference groups are indicated in parentheses. Variables were selected based on clinical relevance and significance in univariate analysis. Multicollinearity was assessed using variance inflation factors, with VIF <2 for all included variables. The logistic regression model demonstrated good fit according to the Hosmer–Lemeshow goodness-of-fit test (*p* = 0.47). *p < 0.05, **p < 0.01, ***p < 0.001.

**Table 4:** Association between comorbidity burden and receipt of disease-specific guideline-concordant standard treatment.

CCI category	Total, *n* (%)	Disease-specific guideline-concordant standard treatment, *n* (%)	Non-standard, de-escalated, or palliative treatment, *n* (%)	P for trend
0–1 (Low)	235 (24.2)	194 (82.6)	41 (17.4)	<0.001^a^
2–3 (Moderate)	428 (44.0)	291 (68.0)	137 (32.0)	
≥4 (High)	309 (31.8)	134 (43.4)	175 (56.6)	
Total	972 (100.0)	619 (63.7)	353 (36.3)	

CCI, Charlson Comorbidity Index. CCI categories were defined as 0–1 (low), 2–3 (moderate), and ≥4 (high comorbidity burden). P for trend was calculated using the Cochran–Armitage trend test. Results indicate a significant inverse association between comorbidity burden and the likelihood of receiving disease-specific guideline-concordant standard treatment.

a Cochran–Armitage trend test.

## Results

### Baseline characteristics of the study population

A total of 972 older patients with gynecologic malignancies were included in the analysis. The mean age was 73.4 ± 5.1 years, with 33.8% aged ≥75 years. Cervical (34.1%), endometrial (36.1%), and ovarian cancer (29.7%) comprised the primary tumor types. Over half (51.3%) were diagnosed at advanced FIGO stages (III–IV). The mean number of comorbidities was 2.9 ± 1.4, and 31.8% of patients had a Charlson Comorbidity Index (CCI) ≥ 4. Hypertension (65.5%), diabetes (40.9%), and coronary artery disease (21.9%) were the most prevalent comorbidities. Regarding functional status, 39.2% had an ECOG performance status ≥2. Overall, 63.7% received disease-specific guideline-concordant standard treatment, while 36.3% received non-standard, de-escalated, or palliative treatment (Table 1).

### Comparison between standard and non-standard treatment groups

Patients receiving non-standard treatment exhibited significantly worse functional and comorbidity profiles compared to those receiving disease-specific guideline-concordant standard treatment. They had higher proportions of advanced FIGO stage (57.5% vs. 47.8%, *p* = 0.002), ECOG ≥2 (62.6% vs. 25.8%, *p* < 0.001), and CCI ≥ 4 (47.3% vs. 22.9%, *p* < 0.001). Additionally, non-standard treatment recipients had a higher burden of chronic conditions, including hypertension, diabetes, coronary artery disease, and COPD (all *p* < 0.001) (Table 2).

### Multivariate predictors of standard treatment receipt

Multivariable logistic regression identified several independent predictors of receiving disease-specific guideline-concordant standard treatment (Table 3). Age ≥75 years (aOR = 0.56, 95% CI: 0.39–0.81), ECOG PS ≥ 2 (aOR = 0.39, 95% CI: 0.28–0.54), CCI ≥ 4 (aOR = 0.52, 95% CI: 0.38–0.72), higher number of comorbidities (per disease: aOR = 0.82), and pulmonary disease (aOR = 0.69) were associated with lower odds of receiving disease-specific guideline-concordant standard treatment. In contrast, higher BMI (aOR = 1.07) and serum albumin levels (aOR = 1.10) were associated with higher odds of receiving disease-specific guideline-concordant standard treatment. No evidence of multicollinearity was observed among the included predictors (all variance inflation factors < 2).

### Association between comorbidity burden and treatment patterns

A significant inverse trend was observed between CCI category and likelihood of receiving disease-specific guideline-concordant standard treatment (P for trend < 0.001). The proportion of patients receiving disease-specific guideline-concordant standard treatment progressively declined from 82.6% in the low CCI group (0–1) to 43.4% in the high CCI group (≥4), supporting an inverse association between comorbidity burden and receipt of disease-specific guideline-concordant standard treatment (Table 4).

### Disease-specific sensitivity analysis by tumor type

To provide a more disease-specific evaluation of the influence of comorbidity burden on treatment allocation, and to address the concern that pooling different gynecologic malignancies could obscure clinically relevant tumor-specific patterns, we performed stratified sensitivity analyses for cervical cancer, ovarian cancer, and endometrial cancer separately. In all three tumor-specific strata, higher comorbidity burden and impaired ECOG performance status showed a consistent direction of association with lower likelihood of receiving disease-specific guideline-concordant standard treatment.

Exploratory stratified analyses showed directionally consistent associations across cervical, ovarian, and endometrial cancer, although the apparent magnitude varied across malignancies and should be interpreted cautiously because of the smaller number of patients and events within each tumor-specific stratum. These differences indicate that tumor type–specific stratification revealed clinically meaningful variation in how comorbidity burden was related to treatment allocation. This finding suggests that a similar level of comorbidity burden may translate into different treatment-allocation consequences depending on the underlying malignancy and its treatment requirements. This pattern is clinically plausible because initial ovarian cancer management often requires deciding between primary cytoreductive surgery and neoadjuvant chemotherapy, a decision that is strongly influenced by patient fitness, surgical feasibility, anticipated cytoreducibility, anesthetic risk, and tolerance of platinum-based chemotherapy. In cervical cancer, the association between comorbidity burden and treatment allocation should be interpreted in the context of FIGO stage and feasibility of radiotherapy-based treatment, including completion of concurrent chemotherapy and brachytherapy, rather than surgical eligibility alone. In endometrial cancer, comorbidity burden may have a less restrictive influence on treatment allocation in some patients because many older patients with early-stage disease can still undergo hysterectomy-based treatment, often through minimally invasive approaches, when perioperative risk is acceptable. These stratified findings support the robustness of the overall host-factor-oriented model while reinforcing that the pooled estimates require disease-specific interpretation. Because subgroup-specific sample sizes were limited, these findings should be interpreted as exploratory evidence of tumor-specific differences in the restrictiveness of comorbidity rather than as definitive comparative effect estimates. This diagnosis-based analysis improved the clinical interpretability of the pooled model by showing that the overall association was not driven by a single tumor type and by revealing clinically plausible differences across malignancies. The detailed stratified results are provided in Supplementary Table S1.

### Model performance and clinical utility

The final predictive model showed favorable discrimination in the internal dataset, with an AUC of 0.933 (95% CI: 0.917–0.948) (Table 5; Figure 1A). The optimal cut-off value (−0.511) yielded a sensitivity of 0.850, specificity of 0.875, and overall accuracy of 0.859. Calibration analysis showed close agreement between predicted and observed probabilities (Figure 1B). Decision Curve Analysis (Figure 1C) demonstrated meaningful net clinical benefit across a threshold probability range of 10–70%. A nomogram was constructed to facilitate individualized clinical prediction based on model coefficients (Figure 1D).

**Table 5:** Discriminative performance and optimal threshold of the predictive model.

Metric	Value
Area Under the Curve (AUC)	0.933
95% Confidence Interval	0.917–0.948
Optimal Cut-off (logit)	−0.511
Sensitivity	0.850
Specificity	0.875
Accuracy	0.859
Youden Index	0.725
Positive Predictive Value (PPV)	0.923
Negative Predictive Value (NPV)	0.769
True Positives (TP)	526
True Negatives (TN)	309
False Positives (FP)	44
False Negatives (FN)	93

AUC, area under the receiver operating characteristic curve; CI, confidence interval; PPV, positive predictive value; NPV, negative predictive value; TP, true positives; TN, true negatives; FP, false positives; FN, false negatives. The dependent variable was receipt of disease-specific guideline-concordant standard treatment. Therefore, true positives indicate patients correctly predicted to receive disease-specific guideline-concordant standard treatment, and true negatives indicate patients correctly predicted not to receive disease-specific guideline-concordant standard treatment. The optimal cut-off point was determined using the Youden Index to maximize combined sensitivity and specificity.

## Discussion

In this retrospective observational cohort study of older women with newly diagnosed cervical, ovarian, or endometrial cancer, comorbidity burden, functional status, age, and nutritional reserve were associated with receipt of disease-specific guideline-concordant standard treatment. Patients with higher CCI scores, impaired ECOG performance status, or advanced age had lower odds of receiving disease-specific guideline-concordant standard treatment, whereas higher BMI and serum albumin levels were associated with higher odds of receiving such treatment. Importantly, the pooled analysis should not be interpreted as suggesting that these malignancies share identical treatment algorithms. Rather, our model was intended to provide a host-factor-oriented decision-support framework that complements disease-specific treatment pathways in real-world geriatric gynecologic oncology practice.

The pooled cohort was used to examine shared host-related factors associated with treatment allocation across major gynecologic malignancies, while recognizing that each cancer type requires disease-specific interpretation. Although cervical, ovarian, and endometrial cancers differ substantially in disease biology and treatment strategies, comorbidity burden and functional impairment are cross-cutting determinants that influence treatment feasibility across cancer types. Therefore, the pooled model was designed to evaluate common host-related factors rather than disease-specific therapeutic choices. This distinction has been clarified throughout the revised manuscript to avoid overgeneralization. We therefore interpret the pooled estimates as reflecting shared host-related determinants of treatment allocation rather than cancer-specific treatment effects. This interpretation is important because direct extrapolation of pooled estimates to a single gynecologic malignancy without considering disease-specific treatment pathways could be misleading. Consistent with this interpretation, we integrated a more granular diagnosis-based evaluation by performing tumor type–stratified sensitivity analyses. These analyses showed directionally similar associations between higher comorbidity burden and lower likelihood of disease-specific guideline-concordant standard treatment across cervical, ovarian, and endometrial cancer, although the strength of association varied by malignancy. These findings support the interpretation that the same level of comorbidity burden may have different practical implications depending on the underlying malignancy and its treatment pathway. Therefore, comorbidity burden should be interpreted within tumor-specific treatment contexts rather than assumed to exert a uniform effect across gynecologic malignancies. The disease-specific interpretation of comorbidity burden can be illustrated by the differing treatment constraints in ovarian, cervical, and endometrial cancer. The ovarian cancer subgroup deserves particular consideration. Unlike many early-stage endometrial cancers, for which hysterectomy-based treatment may remain feasible even in older patients, ovarian cancer frequently requires complex cytoreductive surgery and systemic platinum-based chemotherapy. In this setting, initial treatment allocation is closely linked to the clinician’s assessment of frailty, perioperative risk, tumor burden, resectability, and the likelihood of achieving optimal cytoreduction. Several ovarian cancer-specific tools have been developed to support this process, including laparoscopy-based predictive models for optimal cytoreduction and the laparoscopic risk-adjusted model proposed by Vizzielli et al. (28) to estimate major postoperative complications after primary debulking surgery (29). Therefore, a high comorbidity burden may influence both the probability of receiving disease-specific guideline-concordant standard treatment and the choice between primary cytoreductive surgery and neoadjuvant chemotherapy. In the present study, we did not seek to replace these ovarian cancer-specific surgical feasibility or morbidity-prediction tools; rather, our model provides complementary information on host-related vulnerability that may inform multidisciplinary treatment discussions before applying disease-specific treatment algorithms.

Cervical cancer also requires disease-specific interpretation. In contrast to ovarian cancer, where surgical cytoreduction is central to treatment planning, the role of surgery in cervical cancer is largely stage-dependent and has become more limited in locally advanced disease. For patients with locally advanced cervical cancer, definitive concurrent chemoradiotherapy, usually incorporating external-beam radiotherapy, platinum-based chemotherapy, and brachytherapy when feasible, represents the standard curative-intent approach (23, 24). Therefore, treatment allocation in older patients with cervical cancer should be interpreted with greater attention to FIGO stage, radiotherapy feasibility, ability to tolerate concurrent chemotherapy, feasibility of brachytherapy, treatment duration, baseline organ function, ECOG performance status, and logistical capacity to complete the full radiotherapy course, rather than surgical considerations alone. Accordingly, lack of surgery was not considered non-standard treatment when chemoradiotherapy represented the disease-specific guideline-concordant standard treatment for the patient’s tumor stage.

Endometrial cancer represents another distinct treatment context in which comorbidity may have a different, and potentially less restrictive, impact on treatment allocation. Many patients with endometrial cancer are diagnosed at an early stage, and hysterectomy-based treatment remains the cornerstone of management for apparent uterine-confined disease. In contrast to ovarian cancer, in which optimal cytoreduction may require extensive multivisceral surgery, and cervical cancer, in which locally advanced disease often requires completion of a prolonged chemoradiotherapy course, standard surgery for early-stage endometrial cancer is often limited to total hysterectomy with bilateral salpingo-oophorectomy, with lymph node assessment or sentinel lymph node mapping when indicated. Minimally invasive surgery is increasingly preferred when feasible and may allow many older patients to receive disease-specific guideline-concordant standard treatment despite comorbidity (22, 30). This may explain why comorbidity burden may have a less restrictive influence on treatment allocation in some patients with endometrial cancer than in patients with malignancies requiring more complex cytoreductive surgery or prolonged chemoradiotherapy. Nevertheless, comorbidity, functional status, obesity, cardiopulmonary disease, and anesthetic risk remain clinically relevant when determining whether surgery or adjuvant therapy can be safely delivered. These findings are broadly consistent with previous studies reporting reduced treatment intensity among older or medically complex patients with cancer, but our study provides a more explicit quantitative link between comorbidity burden and disease-specific guideline-concordant standard treatment allocation in older women with gynecologic malignancies. In our cohort, 63.7% of patients received disease-specific guideline-concordant standard treatment, and the proportion decreased markedly from 82.6% in patients with low comorbidity burden (CCI 0–1) to 43.4% in those with high comorbidity burden (CCI ≥ 4). This observed gradient is compatible with the broader oncology literature. For example, Abravan et al. (2) reported that 59.7% of patients in a large pan-cancer cohort had at least one comorbidity at diagnosis. In a Korean retrospective study of older patients with gynecologic cancer, 120 of 251 patients aged ≥65 years did not undergo surgical treatment and were excluded from the surgical cohort, while postoperative complications occurred in 14.5% of those who underwent surgery (4). In ovarian cancer, Assavapokee et al. (5) reported that chemotherapy completion was lower among older patients than younger patients (84.3% vs. 92.6%, *p* = 0.007), and multiple comorbidities were identified as independent factors associated with chemotherapy discontinuation. Compared with these studies, our analysis extends prior evidence by quantifying treatment allocation across CCI categories and by showing that comorbidity burden remained associated with receipt of disease-specific guideline-concordant standard treatment after accounting for tumor type, FIGO stage, and ECOG performance status. However, much of the existing literature either focuses on a single treatment modality or excludes older adults entirely, thereby limiting direct comparability and underscoring the need for disease-specific prospective validation (18).

Importantly, our model utilizes routinely available clinical variables and integrates multiple host-related domains, including comorbidity burden, functional status, and nutritional reserve. The inclusion of BMI and serum albumin—two routinely available indicators associated with nutritional reserve and chemotherapy-related toxicity in older cancer populations—enhances the model’s applicability in low-resource settings, where comprehensive geriatric assessment tools may not be readily accessible (31, 32). Nevertheless, we acknowledge that serum-based markers may be influenced by non-cancer-related conditions such as infection or dehydration, potentially introducing variability into model predictions.

While the model demonstrated strong internal validity (AUC = 0.933), the absence of external validation limits its generalizability. Models derived from single-center retrospective data are susceptible to overfitting, and their performance may deteriorate when applied across different institutions or geographic contexts (33). Although the proportion of missing data was very low (<2%), we acknowledge that complete-case analysis may introduce selection bias if the data were not missing completely at random (MCAR). Therefore, the results of the multivariable models should be interpreted with this consideration in mind. Moreover, treatment decision-making in oncology care for older adults extends beyond clinical indicators; it is often shaped by physician discretion, patient preferences, caregiver involvement, and sociocultural norms—none of which were captured in our dataset.

Clinicians may rely heavily on personal attitudes and assumptions in the absence of robust evidence (34), while family caregivers frequently influence treatment decisions, especially when patients face cognitive or emotional challenges or have limited capacity to comprehend complex medical information (35). Reluctance to pursue aggressive therapy may also stem from concerns over toxicity, diminished independence, or financial burden—factors that warrant incorporation into future prospective models. Another important limitation is the inherent heterogeneity of gynecologic cancers. Cervical, ovarian, and endometrial malignancies differ substantially in disease biology, standard treatment paradigms, radiotherapy requirements, surgical feasibility, and prognostic trajectories. Although tumor type and FIGO stage were considered in the analytic framework, the pooled model should be interpreted as a host-factor-oriented framework rather than a disease-specific treatment algorithm. Residual heterogeneity may still remain because treatment feasibility, radiotherapy requirements, surgical complexity, and patient preferences differ across malignancies and could not be fully captured by tumor type and FIGO stage alone. For cervical cancer, although concurrent chemoradiotherapy and brachytherapy were included in the definition of disease-specific guideline-concordant standard treatment, detailed information on radiotherapy access, brachytherapy feasibility, radiation dose completion, treatment interruptions, concurrent chemotherapy dose intensity, and patient adherence to the full radiotherapy course was not uniformly available; therefore, the model could not fully capture all determinants of radiotherapy feasibility in older patients.

In particular, for ovarian cancer, detailed information required for ovarian cancer-specific assessment tools—such as laparoscopic predictive scores, tumor resectability scores, radiologic tumor burden, ascites volume, surgical complexity, and formal frailty measures—was not consistently available; therefore, we could not incorporate tools such as the Vizzielli score or directly model the decision between primary cytoreductive surgery and neoadjuvant chemotherapy. For endometrial cancer, detailed information on surgical approach, minimally invasive surgery eligibility, sentinel lymph node mapping, anesthesia-specific contraindications, molecular risk classification, and adjuvant treatment indications was not uniformly available; therefore, we could not fully distinguish patients who were poor candidates for hysterectomy-based treatment from those who underwent appropriate guideline-concordant non-surgical or adjuvant strategies. Therefore, our findings should not be used to replace tumor-specific guideline-based decision-making. Future prospective studies should develop and externally validate tumor-specific models that better reflect the clinical nuances, treatment feasibility, and decision-making pathways associated with each cancer type, including ovarian cancer resectability and surgical morbidity assessment, cervical cancer radiotherapy feasibility, and endometrial cancer minimally invasive hysterectomy feasibility. In addition, although tumor type–stratified sensitivity analyses were performed, the sample size within each tumor-specific subgroup limited the precision of subgroup-specific estimates; therefore, these analyses should be considered exploratory and hypothesis-generating. Thus, although stratification reduced the risk of obscuring clinically relevant tumor-specific patterns, residual disease-specific heterogeneity may remain. Accordingly, differences in the apparent restrictiveness of the same comorbidity burden across tumor types should be interpreted cautiously and require confirmation in larger tumor-specific cohorts.

Our findings have several clinical and policy-level implications. First, comorbidity burden should be incorporated into treatment discussions in a structured and transparent manner, rather than being used implicitly as a surrogate for chronological age or as an informal basis for treatment de-intensification. Second, the present model may be most useful as an adjunctive decision-support instrument within multidisciplinary tumor boards, where gynecologic oncologists, medical oncologists, radiation oncologists, anesthesiologists, geriatricians, and nursing teams can jointly evaluate whether older patients remain candidates for disease-specific guideline-concordant standard treatment despite complex medical backgrounds. Third, clinical pathways for older women with gynecologic cancer could incorporate comorbidity burden, ECOG performance status, and nutritional reserve into standardized pretreatment assessment, referral, and shared decision-making processes. As China continues to experience rapid demographic aging, the development and external validation of context-appropriate decision aids for individualized cancer care in older populations should become an important priority.

In summary, this pooled real-world observational cohort study showed that comorbidity burden, functional status, and nutritional reserve were associated with receipt of disease-specific guideline-concordant standard treatment among older women with cervical, ovarian, or endometrial cancer. The internally validated host-factor-oriented model may provide a clinically pragmatic adjunct for supporting discussions about whether patients may remain candidates for disease-specific guideline-concordant standard treatment despite complex medical backgrounds. However, the model should be interpreted within tumor-specific treatment contexts and should not replace disease-specific guideline-based decision-making pathways, including surgical feasibility assessment in ovarian cancer, radiotherapy feasibility assessment in cervical cancer, and hysterectomy or minimally invasive surgery feasibility assessment in endometrial cancer. Further external validation and prospective tumor-specific studies are warranted to assess its generalizability, real-world applicability, and potential impact on treatment equity and outcomes.

## Data Availability

The original contributions presented in the study are included in the article/Supplementary material, further inquiries can be directed to the corresponding authors.
