# Supplementary material for: Association of comorbidity burden with initial treatment allocation in older women with gynecologic cancer: a retrospective cohort study
Source: Front Med (Lausanne). 2026 Jul 28;13:1802265. doi: 10.3389/fmed.2026.1802265 (PMC13457451; doi:10.3389/fmed.2026.1802265)
Supplement: Supplementary file 1 [file Table_1.DOCX]

**Supplementary Table S1.** Tumor type–stratified sensitivity analyses assessing the association between comorbidity burden and receipt of disease-specific guideline-concordant standard treatment.

| **Predictor** | **Cervical cancer** | **Ovarian cancer** | **Endometrial cancer** | **Interpretation** |
| --- | --- | --- | --- | --- |
| High comorbidity burden, CCI ≥4 | Lower likelihood of standard treatment | Lower likelihood of standard treatment | Lower likelihood of standard treatment | Directionally consistent across tumor types |
| ECOG PS ≥2 | Lower likelihood of standard treatment | Lower likelihood of standard treatment | Lower likelihood of standard treatment | Directionally consistent across tumor types |
| Age ≥75 years | Lower likelihood of standard treatment | Lower likelihood of standard treatment | Lower likelihood of standard treatment | Directionally consistent host-related association |
| Higher serum albumin | Higher likelihood of standard treatment | Higher likelihood of standard treatment | Higher likelihood of standard treatment | Directionally consistent favorable association |
| Higher BMI | Higher likelihood of standard treatment | Higher likelihood of standard treatment | Higher likelihood of standard treatment | Directionally consistent favorable association |
| FIGO stage III–IV | Lower likelihood of standard treatment | Lower likelihood of standard treatment | Lower likelihood of standard treatment | Directionally consistent disease-related association |
| Pulmonary disease | Lower likelihood of standard treatment | Lower likelihood of standard treatment | Lower likelihood of standard treatment | Directionally consistent but exploratory association |

**Note:** CCI = Charlson Comorbidity Index; ECOG PS = Eastern Cooperative Oncology Group performance status; BMI = body mass index; FIGO = International Federation of Gynecology and Obstetrics. Stratified analyses were performed separately for cervical cancer, ovarian cancer, and endometrial cancer to evaluate whether the associations observed in the pooled cohort were directionally consistent across tumor types. Because the number of patients and events was smaller within each tumor-specific stratum, these analyses were interpreted as exploratory sensitivity analyses rather than definitive tumor-specific prediction models. This table summarizes the direction of association only; subgroup-specific effect estimates should be interpreted cautiously.
